# A primer on the imaging approach to acute gastrointestinal bleeding

**DOI:** 10.1007/s00261-026-05610-1

**Published:** 2026-06-26

**Authors:** Irfan Amir Kazi, Nanda Deepa Thimmappa, M. Azfar Siddiqui, Sadia Sajid, Emre Demirci, Mohamed Elbanan, Ghassan Hammoud, Ryan Davis, Bareen Waseem Kabir, Aziz Ali Iqbal, Ayesha Nasrullah

**Affiliations:** 1 University of Missouri-Columbia, Columbia, Missouri USA; 2 Asian Institute of Gastroenterology, Hyderabad, India

**Keywords:** Acute gastrointestinal bleeding, Upper gastrointestinal bleeding, Lower gastrointestinal bleeding, CT angiography (CTA), Tc-99m–labeled red blood cell scintigraphy, Catheter angiography

## Abstract

Acute gastrointestinal bleeding (GIB) is a common cause of emergency department presentation, with outcomes ranging from self-limited bleeding to potentially life-threatening hemorrhage. Based on location, acute GIB can be broadly categorized as upper gastrointestinal bleeding (UGIB), lower gastrointestinal bleeding (LGIB), or small-bowel bleeding (SBB). Understanding the clinical presentation and laboratory findings can help determine the appropriate next step in diagnostic evaluation and management. Imaging and endoscopy form the mainstay of evaluation and treatment. Multiphasic CT angiography (CTA), technetium-99 m–labeled red blood cell (RBC) scintigraphy, and catheter angiography are key imaging modalities in the workup of acute GIB. Recognition of common causes, appropriate modality selection, protocol optimization, and awareness of key interpretive pearls and pitfalls are essential for accurate radiologic interpretation and reporting, ultimately improving patient outcomes.

**Supplementary Information:**

The online version contains supplementary material available at https://doi.org/10.1007/s00261-026-05610-1.

## Introduction

Acute gastrointestinal bleeding (GIB) refers to the sudden loss of blood from the gastrointestinal tract and can present with hematemesis, melena or hematochezia [1]. It represents a major healthcare burden in the United States, accounting for over 1 million emergency department visits annually and contributing to approximately 1–2% of all hospitalizations, with over 600,000 hospital admissions each year [2]. The clinical spectrum is broad, ranging from self-limited bleeding to life-threatening hemorrhage, with reported mortality rates between 1% and 10% [3–5]. Although bleeding resolves spontaneously in the majority of patients, up to 20% may experience persistent or recurrent bleeding [6].

A structured, time-sensitive workup and timely management are essential to optimize outcomes in acute GIB. The management in acute GIB is influenced by the underlying etiology, the patient’s clinical presentation, local resource availability for diagnostic evaluation and therapy, and the patient’s response to initial treatment [7]. Imaging plays an integral role in the evaluation and management of acute GIB. Computed tomography (CT), particularly multiphasic CT angiography (CTA), is the primary imaging modality for evaluating acute GIB. Radionuclide imaging and catheter angiography are complementary techniques used in selected cases, with catheter angiography additionally providing a therapeutic option for endovascular hemostasis [8].

This review aims to familiarize radiologists with the clinical presentation, key laboratory findings, and the role of imaging and complementary diagnostic modalities in the evaluation of patients with acute GIB. Common etiologies, practical imaging pearls and pitfalls, and an algorithmic approach to multidisciplinary management are also discussed.

## Clinical presentation

The clinical presentation of acute GIB varies according to the bleeding site, rate and volume of hemorrhage, and underlying etiology. Based on location, bleeding is classified as upper gastrointestinal bleeding (UGIB), originating proximal to the ligament of Treitz (the duodenojejunal flexure), and lower gastrointestinal bleeding (LGIB), originating distal to it [1]. This distinction is important for diagnostic evaluation, management, and risk stratification. UGIB is more common and is generally associated with higher morbidity and mortality than LGIB [4]. In contemporary nomenclature, LGIB is often reserved for bleeding distal to the ileocecal valve (i.e., colonic or rectal sources), whereas hemorrhage distal to the duodenum and proximal to the ileocecal valve is categorized separately as small-bowel (midgut) bleeding [9].

UGIB typically presents with hematemesis, melena, or both. Hematemesis refers to vomiting of bright red blood or “coffee-ground” material, reflecting fresh blood or partially digested blood, respectively. Melena describes black, tarry, foul-smelling stools resulting from the digestion of blood as it transits through the gastrointestinal tract. LGIB typically presents with hematochezia, defined as the passage of bright red or maroon blood per rectum. Exceptions to these typical presentations include brisk, large-volume upper GI bleeding, which may present with hematochezia, and slow bleeding from the small bowel and right colon, which may present with melena. Moreover, the patient’s clinical history and presenting symptoms can provide important clues to the underlying etiology of GIB [1, 7, 10].

## Clinical and laboratory findings

Large-volume acute GIB can lead to tachycardia, hypotension, and orthostatic changes in the blood pressure. It can progress to hypovolemic shock, which may present with cool extremities, weak pulse, oliguria, and altered mental status. A decline in hemoglobin may be observed; however, hemoglobin can be normal early in acute bleeding before intravascular volume equilibration occurs [7, 11]. In acute UGIB, an elevated blood urea nitrogen (BUN) level (> 21–25 mg/dL), and an increased BUN-to-creatinine ratio (> 30:1) may be seen, due to digestion and absorption of blood proteins. Laboratory findings of underlying disease may be seen such as elevated prothrombin time, transaminases or hyperbilirubinemia in chronic liver disease [1, 7, 12].

## Modalities in the work-up of acute GIB

Imaging and endoscopy form the cornerstone of diagnosis and management in acute GIB. The initial workup depends on the suspected location of the bleed (UGIB vs. LGIB), the patient’s clinical status (hemodynamically stable versus unstable), local availability of diagnostic and therapeutic resources, and institutional practice patterns. This review focuses on the major imaging modalities used in acute GIB (CT, radionuclide imaging and catheter angiography) and briefly summarizes the endoscopic techniques relevant to diagnosis and treatment. The salient features of the different imaging modalities are discussed in Table 1. A discussion of diagnostic pathways is presented later in the article.

**Table 1:** Imaging modalities in the evaluation of acute gastrointestinal bleed

	CT angiography (CTA)	Radionuclide imaging (scintigraphy)	Conventional angiography (CA)
Primary role	Diagnostic; active bleed, and often the cause of the bleed	Diagnostic; intermittent or slow bleeds	Therapeutic; intervention (embolization)
Bleeding rate	0.1–0.5 mL/min	0.05–0.2 mL/min	0.5–1.0mL/min
Time and availability	Fast (minutes), widely available	Time-consuming, less available round the clock	Fast (Minutes); Access to interventional radiology can be a limiting factor
Limitations	Cannot detect intermittent/slow bleeding. Contrast risk	Poor anatomic localization, time delay	Invasive, high radiation

### Computed tomography (CT)

CT angiography (CTA) is the imaging modality of choice for evaluation of suspected acute GIB, with a pooled diagnostic performance of 85.2% sensitivity and 92.1% specificity for detecting active hemorrhage in a large meta-analysis [13]. Prior literature suggests that CT angiography can detect bleeding at a rate of 0.5 mL/min. With rapid technical advances and the advent of multislice helical CT scanners, thin-slice reconstructions, and delivery of optimized contrast bolus, newer-generation CT systems may be able to detect active bleeding at rates as low as 0.1 mL/min [9].

The CT angiography examination should be multiphasic, with a non-contrast acquisition followed by late arterial phase imaging (25–35 s post-injection) and venous phase imaging, which can be obtained in the portal venous phase (60–70 s) or the delayed venous phase (70–90 s). Positive oral contrast should not be administered, as it can obscure active bleeding. Neutral contrast is also avoided, as it can delay the scan and can potentially dilute active bleeding, making the diagnosis more challenging. A total of 100–125 mL of iodinated contrast (iodine concentration 350–375 mg/mL) should be administered at a rate of 4–5 mL/s. A bolus-tracking technique is preferred for *arterial phase acquisition* [9, 14, 15]. Using multiplanar reformations and maximum-intensity projection (MIP) images can improve diagnostic yield and can help in the planning of endovascular procedures [15, 16]. A false-negative study may occur in patients with intermittent bleeding who are not actively bleeding at the time of imaging [9].

Dual-energy acquisition should be carried out when available. Virtual non-contrast images can be generated and may obviate the need for a true non-contrast phase, thereby decreasing radiation dose [9]. Using virtual monoenergetic reconstructions at 40–60 keV can improve contrast resolution and, when combined with iodine maps, can increase diagnostic confidence. However, intraluminal hyperdense material (e.g., metallic medications) may sometimes be misclassified as iodine. Therefore, the source images should be reviewed before interpreting bright intraluminal foci on an iodine map as active bleeding [17].

**Fig. 1:**
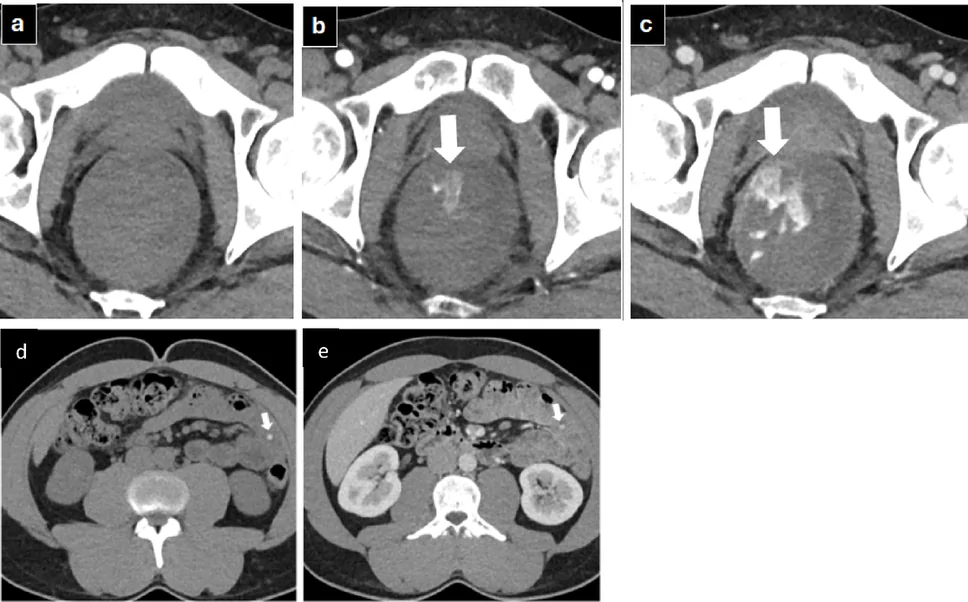
Active GI bleed on a CT angiography study. **a**-**c** Axial precontrast, arterial phase and venous phase images. A focus of arterial contrast extravasation in the rectum is seen in the arterial phase (arrow in image **b**) which expands on the venous phase (arrow in image **c**) and is not seen on the noncontrast phase (image **a**). GI bleed mimic on CT angiography. **d**, **e** Noncontrast and venous phase axial images. A hyperdense focus is seen in a small bowel loop in left lower quadrant on the noncontrast which remains unchanged on the venous phases (arrows)

The primary objective of the CTA is to identify active bleeding. Active arterial bleeding appears as a focus (blush) of contrast on the arterial phase that increases in size on the venous phase and is absent on the non-contrast images (Fig. 1a-c). Venous bleeding, or slow arterial bleeding, may be visualized only in the venous phase [9, 18]. In some cases, active bleeding may not be directly identified but localized intraluminal hyperattenuating bowel contents (sentinel clot sign) may help localize the bleeding site (Fig. S1a and b), with the caveat that intraluminal contents can migrate due to peristalsis [16]. Conversely, the presence and location of a bowel wall hematoma can help localize the bleeding source, and the involved bowel segment should be closely interrogated to identify the site of active bleeding [19].

The non-contrast phase is essential because, without it, hyperattenuating intraluminal debris or material may be mistaken for active bleeding (Fig. 1d and e) [18]. The best way to determine whether intraluminal material represents blood products is to measure its attenuation. Blood products may also appear relatively hyperattenuating compared with other bowel contents when narrow window width settings (e.g., brain window) are used (Fig. S1 a and b). On non-contrast images, intraluminal blood products typically measure 30–80 Hounsfield units (HU). Some studies have reported attenuation values up to 90 HU, and material with attenuation above this range on a non-contrast CT is unlikely to represent blood products [9, 20]. Hyperattenuating intraluminal contents can include previously administered positive oral contrast (iodinated or barium), medications containing metals (e.g., calcium, magnesium, aluminum, or bismuth), ingested foreign bodies (e.g., bones, certain seeds, and metal), clips and suture material, and hyperattenuating fecal material [15, 17, 21]. On the post-contrast phase, hyperenhancing bowel mucosa, hypervascular bowel lesions and vascular pathologies can be mistaken for active contrast extravasation [19]. The suspected focus of bleeding identified on the arterial phase should demonstrate expansion on the venous phase. This is important for two reasons. First, intraluminal contents can shift due to peristalsis and may not be seen in the same location on the non-contrast phase as on the arterial phase, leading to a false-positive examination (Fig. S2 a-d). Second, arterial lesions such as aneurysms or pseudoaneurysms typically do not increase in size on the venous phase, unlike active contrast extravasation [19]. An exception is that venous malformations may become more conspicuous on delayed-phase imaging.

Beyond identifying active bleeding or intraluminal blood products, CT also plays a key role in determining the underlying etiology. Often, active hemorrhage or intraluminal blood products are not demonstrated, yet the cause of bleeding is evident. In addition to looking for active bleeding and blood products, attention should be paid to the bowel wall for abnormal thickening or enhancement. The mesenteric vasculature should be examined for vascular abnormalities. Utilizing MIP images, particularly coronal projections, can help delineate subtle findings [15, 19].

Limitations of CT angiography include exposure to ionizing radiation and the need for intravenous contrast administration [8]. Strategies to reduce radiation dose include performing a low-dose unenhanced acquisition and utilizing dual-energy CT for generating virtual non-contrast images when available. Utilizing split-bolus contrast technique, instead of acquiring separate arterial and venous phase images, has also been proposed [8, 9]. Nevertheless, the primary objective of the study is to accurately diagnose active bleeding, and this should remain the focus, as suboptimal evaluation can decrease diagnostic confidence. Intravenous contrast administration may predispose to contrast-induced nephropathy in patients with low glomerular filtration rate (GFR), although this can be confounded by acute renal failure in the setting of hypovolemic shock due to blood loss. There is also a very small risk of severe allergic reactions. Furthermore, unlike endoscopy and catheter angiography, CT is primarily a diagnostic modality and does not allow therapeutic intervention [15].

### Radionuclide imaging

Technetium-99 m–labeled red blood cell (RBC) scintigraphy is the radionuclide study of choice for evaluation of acute gastrointestinal hemorrhage. Active bleeding at rates of 0.05–0.2 mL/min can be detected. It is most appropriate for evaluation of intermittent or slow bleeding. Reported sensitivity and specificity are as high as 93% and 95%, respectively [22].

Images are acquired using a large field-of-view gamma camera, and approximately 20 mCi of technetium-99 m–labeled RBCs are administered intravenously. Initial flow-phase images are acquired for 60 s at 1–3 s per frame, followed by dynamic imaging for up to 90 min at 1 min per frame. A frame rate of 1 frame per 10–20 s improves localization of the gastrointestinal bleeding site by accounting for rapid bowel peristalsis and movement of intraluminal blood. In contrast, slow bleeding may be less conspicuous at faster frame rates and can be better identified by reconstructing images into longer frames [23]. When no bleeding site is identified within 60–90 min, delayed imaging can be obtained up to 24 h post-injection at 1 min per frame with no additional radiation exposure.

**Fig. 2:**
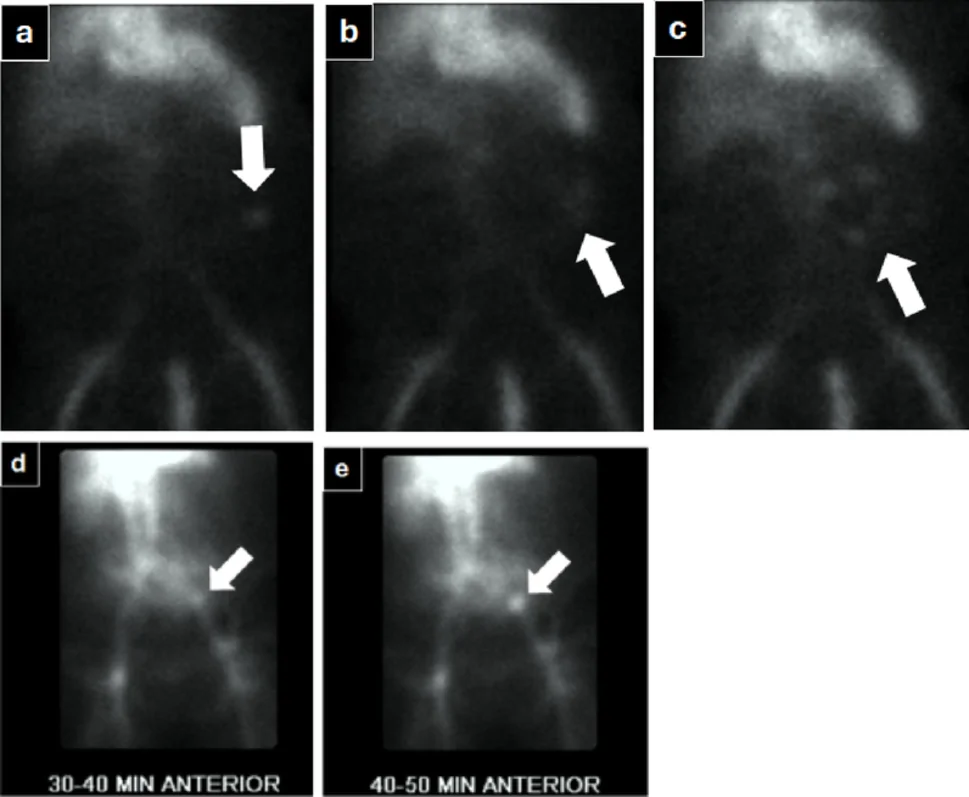
Active gastrointestinal bleeding on Tc-99 m–labeled RBC scintigraphy. Serial 1-min images **a**–**c** from a 60-min dynamic anterior abdominal acquisition demonstrate a focus of radiotracer extravasation in the right upper quadrant, first seen approximately 24 min after injection of the radiolabeled RBCs (not shown). The focus progressively increased in intensity (arrow, a) and subsequently demonstrates dynamic movement within the bowel lumen (arrows, **b** and **c**), confirming active gastrointestinal bleeding. The localization suggests bleeding likely originating from the proximal jejunum. GI bleeding mimic on Tc-99 m–labeled RBC scintigraphy. **d**, **e** Summed static images from the anterior abdominal dynamic acquisition demonstrate a focal area of abnormal tracer uptake in the mid-left abdomen. The focus shows no dynamic movement typical of gastrointestinal bleeding and remained stable on delayed imaging (not shown). This corresponded to the patient’s known mesenteric pseudoaneurysm (not shown)

Active bleeding is identified as radiotracer activity outside the expected blood pool that focally increases in intensity and demonstrates antegrade movement, retrograde movement, or both within the bowel lumen over time (Fig. 2a-c). The source of gastrointestinal bleeding should be identified based on the first site where activity appears, rather than the area showing the greatest intensity, size, or most proximal distribution. Multiple other conditions can show focal uptake including gastric uptake, renal pelvic activity, ectopic kidney, ureteral activity, transplanted kidney, accessory spleen, vascular conditions such as varices, angiodysplasia, and aneurysms [24]. SPECT/CT can help differentiate abdominal tracer uptake related to anatomic variants or physiological processes from true occult gastrointestinal bleeding (Fig. 2d, e) [23].

While it is typically possible to distinguish small bowel from large bowel bleeding, accurate anatomic localization remains limited and may be enhanced using SPECT/CT. Other limitations include the time-consuming nature of the examination, especially when no definite bleeding focus is identified. This is a limiting factor in hemodynamically unstable patients. Patient-related factors that can affect red blood cell labeling include low hemoglobin, hemoglobinopathies, recent transfusion, and certain medications (e.g., heparin) [22, 24]. However, modern labeling techniques are less susceptible to interference from medications and iodinated contrast agents [23].

### Catheter angiography (CA)

In contrast to CT and radionuclide studies, CA serves both diagnostic and therapeutic purposes, although it is primarily used for management. Catheter angiography can detect bleeding at rates of approximately 0.5–1.0 mL/min [15]. When active bleeding is identified, precise localization of the culprit vessel is possible. Diagnostic performance is variable, with reported sensitivity of approximately 60% for detecting acute gastrointestinal bleeding, with specificity close to 100% [25, 26]. CA is rarely performed as the primary diagnostic test for acute GIB because CTA has superior diagnostic capability and provides a roadmap for subsequent CA [9]. Limitations include its invasive nature, the time-consuming nature of the procedure, and the potential for exposure to a high radiation dose. The study may be falsely negative in cases of intermittent gastrointestinal bleeding [6, 25].

CA for intervention typically involves selective angiography of the celiac and superior mesenteric arteries in upper GIB, and the superior and inferior mesenteric arteries in lower GIB. Identification of contrast extravasation confirms the bleeding site and allows treatment with embolization (e.g., microcoils or glue). Embolization is performed after superselective cannulation of the end vessels (vasa recta) supplying the involved bowel segment to achieve hemostasis while preserving collateral perfusion and thereby minimizing the risk of ischemia. When a bleeding source identified on CT or endoscopy is not demonstrated on CA, prophylactic embolization of the suspected bleeding vessel may be considered [9].

### Other modalities in evaluation of acute GIB

Esophagogastroduodenoscopy (EGD) and colonoscopy remain the cornerstone modalities for both diagnosis and management of acute upper and lower gastrointestinal bleeding respectively, given their high diagnostic yield and ability to provide immediate therapeutic intervention. In cases of suspected small-bowel bleeding, particularly when initial upper and lower endoscopic evaluations are negative, capsule endoscopy serves as a valuable noninvasive diagnostic tool for identifying mucosal sources of bleeding. Capsule endoscopy is particularly useful in hemodynamically stable patients with obscure or intermittent bleeding; however, its use is limited in the acute setting due to the inability to provide therapeutic intervention and the potential for delayed localization. Importantly, capsule endoscopy is contraindicated in patients with known or suspected gastrointestinal obstruction, strictures, or fistulas, due to the risk of capsule retention. While conditions such as inflammatory bowel disease, prior abdominal radiation, and previous bowel surgery may increase this risk, they are considered relative contraindications and warrant careful patient selection and, when appropriate, prior evaluation with cross-sectional imaging or patency capsule testing [9, 27].

When available, device-assisted enteroscopy techniques, such as single or double-balloon enteroscopy, allow for direct visualization of small-bowel pathology and offer the significant advantage of enabling therapeutic interventions, including hemostasis, biopsy, and polypectomy. These techniques are particularly valuable following lesion localization by capsule endoscopy or imaging studies, facilitating targeted evaluation and management [28].

### Other imaging modalities

CTA, radionuclide imaging, and CA form the mainstay of evaluation in overt acute GIB, which presents with hematemesis, melena, or hematochezia. Occult GIB does not present with these symptoms and is diagnosed in the presence of a positive stool test or when there is unexplained iron-deficiency anemia. CT enterography can aid in the diagnostic workup of overt GIB with a negative initial evaluation and in cases of occult GIB when small-bowel pathology is suspected. MR enterography can be helpful for assessing suspected inflammatory causes of small-bowel GIB [9, 15]. However, CT enterography and MR enterography are not considered first-line studies in overt acute GIB because they are not intended for rapid evaluation of active hemorrhage in the acute setting and are primarily problem-solving examinations in hemodynamically stable patients after negative initial workup [9, 29, 30]. These examinations are not discussed in detail in the present review, as they are not routinely performed for the evaluation of acute overt GIB.

A Tc-99 m pertechnetate scan (Meckel’s scan) can be performed when Meckel’s diverticulum is suspected as a cause of acute GIB; however, this study should not be performed when the patient is actively bleeding as active bleeding can dilute or obscure tracer localization, introduce competing intraluminal activity, and reduce diagnostic accuracy. A Tc-99 m pertechnetate scan is a targeted examination performed when Meckel’s diverticulum is specifically suspected, rather than a general first-line test for overt acute GIB [18, 31].

## Causes of acute GIB

In the present review, acute GIB is broadly categorized as bleeding due to vascular pathologies or due to nonvascular pathologies with secondary hemorrhage. These are summarized in Table 2. Common causes of acute GIB are listed in Table 3.

**Table 2:** Classification of causes of acute gastrointestinal bleed

Category	Subcategory	Specific causes
Vascular pathologies	Vascular malformations	Arteriovenous malformations (AVMs); Pseudoaneurysm; Varices; Hemorrhoids; Dieulafoy malformation; Angiodysplasia; Gastric antral vascular ectasia (GAVE); Syndromic
	Other vascular causes	Aortoenteric fistula; Hemobilia/hemosuccus pancreaticus; Ischemic colitis; chronic radiation proctitis/enteritis
Non-vascular pathologies with secondary hemorrhage	Inflammatory	Crohn’s disease; Ulcerative colitis; acute radiation proctitis/enteritis
	Infective	*E. coli*; *Shigella*; *Entamoeba histolytica*; Viral
	Erosive/ulcerative	Mallory–Weiss tear; Erosive gastritis/esophagitis; Peptic ulcer disease; Anal fissure; Drug-induced (NSAIDs, aspirin, steroids)
	Coagulopathy	Anticoagulant use; Hemophilia; Disseminated intravascular coagulation (DIC); Thrombocytopenia/platelet dysfunction
	Masses	· Benign · Malignant
	Trauma/iatrogenic	Accidents; Foreign body; Post-surgery/post-endoscopy
	Diverticular disease	Meckel’s diverticulum; Colonic diverticulosis; Small-bowel diverticulosis

**Table 3:** Common causes of acute gastrointestinal bleed

Acute upper gastrointestinal bleeding	Peptic ulcer disease (40–50%)
	Gastritis and duodenitis (25–30%)
	Esophageal variceal bleeding (5–10%)
	Less common causes (10–12%)- Dieulafoy lesion, Mallory Weiss tears, Malignancy
Acute lower gastrointestinal bleeding	Diverticular disease (23–50%)
	Ischemic colitis (6–16%)
	Hemorrhoids (4–20%)
	Colorectal neoplasms (2–15%)
	Angioectasia (5–10%)
	Rectal ulcer, post-polypectomy, radiation proctitis, inflammatory and infective colitis
Acute small bowel bleeding	Vascular abnormalities (middle aged and elderly)
	Inflammatory bowel disease (young adults)
	Small bowel tumors
	Meckel’s diverticulum
	Dieulafoy lesion, angioectasia

## Vascular pathologies implicated in the etiology of acute GIB

Portal hypertension in the setting of chronic liver disease leads to the formation of portosystemic venous collaterals, resulting in the development of varices (Fig. S3). More than 60% of GIB cases in patients with chronic liver disease occur due to esophageal varices, followed by gastric varices (5–10%) [32]. Less common causes include ectopic varices, which are portosystemic venous collaterals involving portions of the gastrointestinal tract other than the esophagus or stomach. Portal hypertensive gastropathy, enteropathy, and colopathy can also predispose to acute GIB. In addition to identifying bleeding, CT can also demonstrate portosystemic venous collaterals and other complications of chronic liver disease and provide a roadmap for further management [33, 34]. In many cases, chronic liver disease may not be known at presentation, and imaging can help identify underlying chronic liver disease as the cause of bleeding [33, 35].

**Fig. 3:**
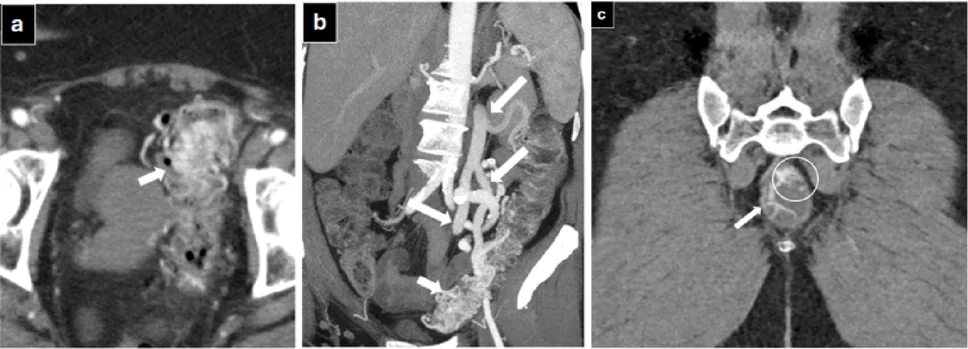
Angiodysplasia of the colon in a 56-year-old woman. CT angiography was performed for evaluation of acute GI bleeding. **a** Axial and **b** coronal MIP arterial phase images demonstrate a cluster of vessels in the distal descending and proximal sigmoid colon (short arrows), with early filling of the inferior mesenteric vein and its tributaries (long arrows). No active bleeding was identified on CT. Active bleeding was subsequently confirmed on colonoscopy. Rectal bleeding secondary to Dieulafoy malformation. **c** Coronal venous phase CT image demonstrates prominent rectal vasculature (arrow) with a focus of active contrast extravasation (circle). Colonoscopy confirmed a Dieulafoy vascular malformation with active bleeding

Gastrointestinal vascular malformations account for about 5% of acute GIB. However, they constitute a disproportionately higher proportion of small-bowel bleeding, accounting for about 30–40% of small-bowel hemorrhage [36]. Common vascular malformations include angiodysplasia (angiectasia), gastric antral vascular ectasia (GAVE), Dieulafoy malformation, and arteriovenous malformations. Angiodysplasias are caused by dilated vessels (veins or capillaries) in the bowel wall, with a prominent feeding artery and an early draining vein due to vascular shunting (Fig. 3a, b) [37, 38]. Angiodysplasias occur in the elderly population and are associated with aortic stenosis (Heyde syndrome), chronic kidney disease, von Willebrand disease, and use of a left ventricular assist device [36]. Dieulafoy malformation accounts for about 1–2% of acute GIB and is characterized by a prominent submucosal artery; it is most commonly seen in the stomach (70%). This condition can be difficult to diagnose on imaging in the absence of active hemorrhage due to the small size of the artery. Since it is an arterial malformation, it can cause brisk hemorrhage (Fig. 3c) [39].

Arteriovenous fistulas and arteriovenous malformations are other types of vascular malformations characterized by high intralesional flow, with a prominent feeding artery and an early draining vein (Fig. S4). Syndromic vascular malformations can occur in hereditary hemorrhagic telangiectasia and blue rubber bleb nevus syndrome(Fig. S5) [37, 38]. Pseudoaneurysms are a rare cause of GIB. Pseudoaneurysms typically arise secondary to pancreatitis, hepatobiliary, gastric or pancreatic surgery, peptic ulcer disease or trauma (Fig. S6). The gastroduodenal artery is a common site of pancreatitis-related pseudoaneurysm and can cause severe GI bleeding (Fig. S7) [40, 41].

Hemosuccus pancreaticus and hemobilia are rare causes of acute gastrointestinal bleeding. Hemosuccus pancreaticus refers to GI bleeding due to hemorrhage into the pancreatic duct, most commonly occurring as a complication of acute or chronic pancreatitis (Fig. S7). Hemobilia is most commonly secondary to trauma, malignancy, and post-procedural or post-interventional injury [42]. Ischemic colitis, the second most common cause of lower GIB, occurs secondary to vascular insufficiency of the colon and is associated with arterial occlusion, emboli, and arterial hypoperfusion/insufficiency. It typically presents with abdominal pain and bloody stools. Imaging findings include bowel wall thickening, mural stratification, and thumbprinting, often with a segmental or territorial distribution (Fig. S8) [43]. Aortoenteric fistula is a rare cause of acute GIB.

## Nonvascular pathologies with secondary hemorrhage causing acute GIB

These conditions can be further subdivided based on the cause of bleeding. Erosive or ulcerative conditions include Mallory–Weiss tear, erosive gastritis or esophagitis, peptic ulcer disease commonly caused by H. pylori infections or drugs (NSAIDs, aspirin, steroids), and anal fissure. Peptic ulcer disease is the most common cause of upper GIB, accounting for approximately half of cases of upper GIB (with higher proportions reported in non-variceal UGIB cohorts) (Fig. S9) [25, 44].

Common infections that can cause acute GIB include *Escherichia coli*, *Shigella*, *Salmonella*,* Clostridioides difficile*,* Entamoeba histolytica*, and viral enteritis [45]. Inflammatory conditions include ulcerative colitis, Crohn’s disease, and acute radiation enteritis. Crohn’s disease is among the most common causes of small bowel bleeding in young adults [9]. Coagulopathy can also cause acute GIB, with etiologies including anticoagulant use, hemophilia, disseminated intravascular coagulation, cirrhosis, thrombocytopenia, and platelet dysfunction (Fig. S10) [46–48].

**Fig. 4:**
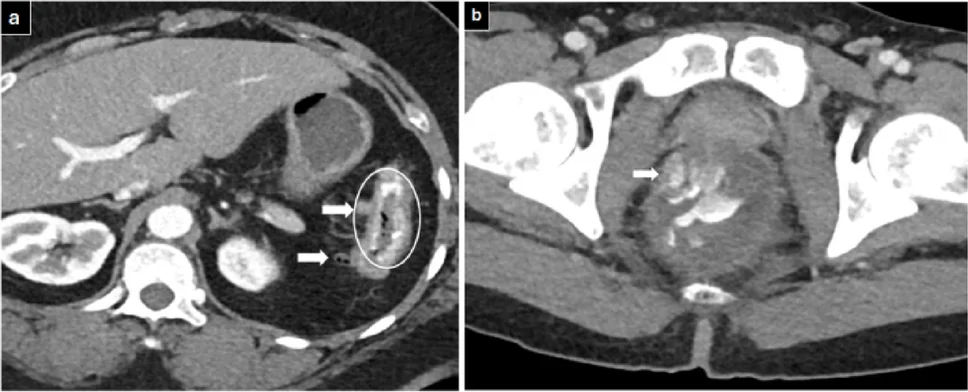
Colonic diverticular bleeding. **a** Axial venous phase CT image demonstrates active contrast extravasation in the region of the splenic flexure, initially seen on the arterial phase (not shown) and expanding on the venous phase (circle). Extensive background colonic diverticular disease is also present (arrows). The bleeding resolved spontaneously. Colonoscopy demonstrated extensive diverticular disease and sequelae of prior hemorrhage, without evidence of active bleeding. Active rectal bleeding secondary to rectal wall laceration from foreign body insertion. **b** Axial venous phase CT image demonstrates active contrast extravasation within the rectum (arrow). Colonoscopy identified a laceration along the anterior rectal wall, which was treated with placement of an Endo Clip

Acquired diverticular disease most commonly involves the colon, although it may also occur in the duodenum and, less commonly, in the jejunum or ileum. Hemorrhage is a recognized complication. Colonic diverticular disease is the most common cause of lower GIB and accounts for up to 50% of cases in some series (Fig. 4a) [18, 49, 50]. Symptomatic Meckel’s diverticulum containing ectopic gastric mucosa typically presents with lower gastrointestinal bleeding in the pediatric and young adult population. It is the most common cause of severe, painless lower GIB in children (Fig. S11) [18].

Tumors can also cause acute GIB, commonly due to fragile tumor neovascularity or vascular invasion. Tumors include gastric, small-bowel, and colonic adenocarcinomas; gastrointestinal stromal tumors; neuroendocrine tumors; metastases; and solid organ tumors invading the bowel. Rarely, benign tumors can also cause acute GIB (Fig. S12) [51–54]. Post-procedural acute GIB can occur as a sequela of bowel surgery at the anastomotic site or after endoscopic interventions such as polypectomy (Fig. S1) [7, 55]. Other rare causes include blunt and penetrating trauma and foreign bodies (Fig. 4b) [56, 57]. Occasionally, hemoptysis or brisk bleeding from the oral cavity or pharynx may be mistaken for gastrointestinal bleeding, and imaging can help localize the true source of hemorrhage. Sometimes, acute GIB is diagnosed with imaging but the etiology for the bleed is not identified [19].

## Algorithmic approach to acute gastrointestinal bleeding

The workup of overt gastrointestinal bleeding is tailored to the clinical presentation and whether the bleeding is suspected to arise from the upper or lower gastrointestinal tract, as this distinction influences subsequent diagnostic and management decisions. The algorithmic approach is illustrated in Fig. S13.

### Approach to suspected acute UGIB

Esophagogastroduodenoscopy (EGD) is the first-line modality for evaluation of suspected acute UGIB, owing to its high diagnostic accuracy and the ability to provide immediate therapeutic intervention, including injection therapy, thermal coagulation, mechanical clipping, and variceal band ligation. In hemodynamically stable patients, early endoscopy, typically within 24 h of presentation, is recommended after appropriate resuscitation and risk stratification, as it improves diagnostic yield and clinical outcomes.

Multiphasic CTA plays an important complementary role, particularly when endoscopy is unavailable, contraindicated, or nondiagnostic (e.g., negative endoscopy despite ongoing clinical suspicion of bleeding). CTA is especially valuable in hemodynamically unstable patients with suspected active bleeding. CA has a limited role in diagnosing bleeding in patients in whom active bleeding is not identified on CTA. Its primary role is therapeutic intervention in patients with active bleeding demonstrated on CTA, when endoscopy is unsuccessful, or in cases of recurrent bleeding [9, 29, 58, 59].

### Approach to suspected acute LGIB

Multiphasic CTA is the imaging modality of choice in hemodynamically unstable patients and in hemodynamically stable patients with a high suspicion for active bleeding. CTA enables rapid detection and localization of active hemorrhage and may identify the underlying etiology, thereby facilitating timely triage to endovascular or surgical intervention. Colonoscopy is generally the first-line diagnostic and therapeutic modality in stable patients, as it allows for direct visualization, localization, and treatment of the bleeding source in a single session. A key limitation is the need for bowel preparation, which can be a major constraint in the emergent setting [9, 30, 46].

CA can be performed for therapeutic intervention in patients with active bleeding identified on CTA, particularly in hemodynamically unstable patients and in those in whom colonoscopy has failed to identify or control the bleeding source. Transcatheter embolization allows for targeted treatment of the bleeding vessel, with high technical success rates. However, similar to other modalities, its sensitivity may be limited in the setting of intermittent bleeding [9, 60].

Technetium-99 m–labeled RBC scintigraphy serves as a complementary modality, particularly in cases with intermittent or slow bleeding that may not be detected on CTA or colonoscopy. It is often utilized following negative or inconclusive CTA and colonoscopy or when these modalities are unavailable or contraindicated. Although highly sensitive for low-rate bleeding, its clinical utility is limited by prolonged acquisition times and relatively poor anatomic localization, which may necessitate further imaging or intervention for definitive management [9, 30].

### Approach to suspected acute bleed from the small bowel

Imaging plays a vital role in the evaluation of suspected acute small-bowel bleeding, as conventional endoscopic modalities such as EGD, colonoscopy, and push enteroscopy do not adequately visualize most of the small intestine. Consequently, cross-sectional and angiographic imaging techniques form the backbone of diagnosis and management in the acute setting. Multiphasic CTA is typically the preferred initial modality in patients with suspected active small-bowel bleeding, particularly in those who are hemodynamically unstable or have ongoing overt hemorrhage. CTA enables rapid detection and localization of active extravasation, may identify the underlying etiology (e.g., vascular malformations, tumors, or inflammatory pathology), and provides a vascular roadmap to guide subsequent CA and intervention.

In hemodynamically stable patients without evidence of brisk bleeding, CT enterography and capsule endoscopy function as important problem-solving tools for identifying mucosal and submucosal sources of bleeding. Capsule endoscopy is especially valuable in the evaluation of obscure small-bowel bleeding following negative upper and lower endoscopy, while CT enterography provides superior assessment of mural and extraluminal pathology. When a lesion is identified or strongly suspected, device-assisted enteroscopy (e.g., single or double-balloon enteroscopy) can be employed for definitive diagnosis and therapeutic intervention [9, 25, 37, 58].

## Key points

### CT angiography (CTA)

- Enhancing bowel loops, varices, vascular structures, calcifications, or ingested material may mimic active bleeding; a multiphasic CTA protocol (noncontrast, arterial, and venous phases) helps reduce false-positive interpretation.
- Noncontrast images are essential for distinguishing pre-existing hyperdensities (e.g., barium or ingested material) from true active bleeding.
- Arterial and venous phase images should be compared carefully; progressive pooling or enlargement of contrast supports active bleeding.
- Intermittent bleeding may be missed on CTA; repeat CTA or Tc-99 m–labeled RBC scintigraphy should be considered when clinical suspicion remains high.
- In many cases, CT can also identify the underlying cause of bleeding, including vascular malformations, inflammation, or malignancy.

### Transcatheter angiography

- Typically performed after a positive CTA or in the setting of massive, ongoing bleeding.
- Angiographic confirmation of cessation of bleeding after embolization is important to ensure technical success.

### Tc-99 m–labeled RBC scintigraphy

- Best suited for intermittent or slow-rate bleeding.
- Distal movement of tracer supports intraluminal gastrointestinal bleeding.
- Anatomic localization may be challenging; positive studies should be correlated with CTA or other imaging for precise localization.

### Role of multidisciplinary management

- Overreliance on imaging may delay definitive management; some patients may require urgent endoscopy as the first step.
- Imaging findings should always be interpreted in conjunction with hemodynamic status and overall clinical stability when planning diagnostic or therapeutic management.

## Conclusion

Imaging plays a central and increasingly pivotal role in the diagnosis and management of acute gastrointestinal bleeding, serving as both a rapid localization tool and a guide for definitive therapy. A thorough understanding of the clinical presentation, underlying etiologies, and strengths and limitations of available imaging and complementary modalities is essential for selecting the most appropriate diagnostic pathway. Close collaboration among radiologists, gastroenterologists, interventional radiologists, and surgeons is critical to optimize patient outcomes.

## Supplementary Information

Below is the link to the electronic supplementary material.

Supplementary Material 1

## Data Availability

No datasets were generated or analysed during the current study.
