# Supplementary material for: A primer on the imaging approach to acute gastrointestinal bleeding
Source: Abdom Radiol (NY). 2026 Jun 26;51(12):6309–19. doi: 10.1007/s00261-026-05610-1 (PMC13768375; doi:10.1007/s00261-026-05610-1)
Supplement: Supplementary file 1 [file 261_2026_5610_MOESM1_ESM.docx]

**Figure S1:** **An 88-year-old man with acute gastrointestinal bleeding at the anastomotic site after extended left hemicolectomy.** A noncontrast CT was performed to evaluate postoperative bleeding in the setting of declining hemoglobin and acute kidney injury. **(a, b)** Coronal noncontrast CT images demonstrate hyperattenuating intraluminal contents at the anastomotic site (vertical long arrow) and in the ascending colon (horizontal long arrow). In comparison, simple fluid attenuation contents are seen in the stomach and small bowel (short arrows). This difference is more conspicuous on narrow window settings in image **b**. A curved arrow indicates an associated extramural hematoma adjacent to the anastomotic site. The hyperattenuating intraluminal contents are densest at the anastomotic site, suggesting this as the bleeding source, consistent with the sentinel clot sign. It subsequently progressed to an anastomotic dehiscence, which was managed with surgical repair and creation of a diverting loop ileostomy.

**Figure S2. GI bleeding mimic on CT angiography.** **(a, d)** Noncontrast, **(b)** arterial phase, and **(c)** venous phase coronal images. A hyperdense focus is seen within a small-bowel loop in the left lower quadrant on the arterial phase image (arrow) and remains unchanged on the venous phase image (arrow). Because the focus did not evolve on the venous phase compared with the arterial phase, it was favored not to represent active bleeding. However, because the focus was not initially identified on the noncontrast phase, it posed a diagnostic challenge. Careful review showed that the hyperdense focus had shifted on the arterial phase compared with the noncontrast phase due to peristalsis; on the noncontrast image, it was located in an adjacent small-bowel segment (**d**, arrow).

**Figure S3. Upper gastrointestinal bleeding from varices secondary to portal hypertension in the setting of chronic liver disease.** Upper gastrointestinal endoscopy and contrast-enhanced CT (CECT) of the abdomen demonstrated large gastric fundal varices with a gastrorenal shunt (GRS). **(a)** Axial CECT MIP image shows large fundal varices (arrow). The liver demonstrates a lobulated contour and hypertrophy of the left lateral segment, consistent with morphologic changes of chronic liver disease. **(b)** Coronal CECT MIP image shows large gastric fundal varices with a gastrorenal shunt (arrows). **(c)** The GRS was accessed via the right common femoral vein, and a 22-mm vascular plug was placed within the shunt with a catheter advanced across the plug (arrow). **(d)** Sclerosant was injected through the catheter, opacifying the varices (arrow). The plug was deployed after complete filling of the varices.

**Figure S4. Acute gastrointestinal bleeding secondary to a jejunal arteriovenous malformation.** The patient presented with melena. **(a, b)** Axial and coronal arterial phase CT angiography images demonstrate the jejunal AVM (arrows). **(c)** The SMA branch supplying the AVM was superselectively cannulated, and DSA demonstrates filling of the AVM with an early draining vein (arrow). Embolization was performed using a glue and lipiodol mixture. **(d)** Post-embolization image demonstrates no residual filling of the AVM (arrow).

**Figure S5. Upper gastrointestinal bleeding in the setting of blue rubber bleb nevus syndrome.** **(a, b)** Axial and coronal venous phase CT images demonstrate prominent veins at the duodenojejunal flexure (circle), which were not apparent on the arterial phase images (not shown). No active contrast extravasation was identified on CT. Tc-99m–labeled RBC scintigraphy was also negative for hemorrhage. Active bleeding was subsequently identified on capsule endoscopy. Surgical resection confirmed dilated submucosal veins in this region.

**Figure S6. Pseudoaneurysm of a left gastric artery branch causing acute gastrointestinal bleeding.** **(a)** Coronal MIP contrast-enhanced CT image shows no obvious vascular abnormality. **(b)** Left gastric artery angiogram demonstrates a pseudoaneurysm arising from a branch of the left gastric artery with active contrast extravasation. **(c)** Contrast extravasation is seen within the stomach (arrow). **(d)** Glue embolization of the pseudoaneurysm and the supplying branch was performed. The patient was a 9-year-old girl who presented with shock and upper gastrointestinal bleeding following EUS-guided liver biopsy.

**Figure S7. Hemosuccus pancreaticus caused by a gastroduodenal artery pseudoaneurysm.** The patient was a 35-year-old man who presented with acute pancreatitis and acute gastrointestinal bleeding. Endoscopy demonstrated hemosuccus pancreaticus. **(a)** Coronal arterial phase CT angiography image shows a pseudoaneurysm arising from a branch of the gastroduodenal artery (arrow). **(b)** The gastroduodenal artery was selectively cannulated, and DSA demonstrates the pseudoaneurysm (short arrow) with active contrast extravasation (long arrow). **(c)** Embolization was performed using a glue and coil combination. **(d)** Post-embolization image demonstrates no residual filling of the pseudoaneurysm.

**Figure S8. Ischemic colitis at the SMA–IMA watershed territory (Griffiths’ point).** The episode occurred 2 weeks after aortic valve replacement. Reformatted coronal postcontrast venous phase CT image demonstrates wall thickening in the distal transverse colon with mural stratification and perienteric fat stranding (short arrows). The proximal transverse colon and the mid and distal descending colon appear normal (long arrows). The mesenteric arterial and venous vasculature were patent (not shown). Findings were favored to represent ischemic colitis, likely related to an embolic event following recent valve replacement.

**Figure S9. Duodenal bulb ulcer.** Contrast-enhanced CT (CECT) of the abdomen was performed for evaluation of acute gastrointestinal bleeding and a drop in hemoglobin. **(a, b)** Axial and coronal CECT images demonstrate a focal outpouching in the duodenal bulb with adjacent inflammatory changes (arrows). No active bleeding was identified on imaging. Endoscopy subsequently demonstrated a bleeding duodenal bulb ulcer.

**Figure S10. Cecal bleeding after heparinization and antiplatelet therapy for ST-elevation myocardial infarction.** **(a, b)** Axial arterial and venous phase CT images demonstrate a focus of contrast extravasation in the cecum on the arterial phase that enlarges on the venous phase, consistent with active bleeding (arrows).

**Figure S11. Meckel’s diverticulum.** The patient presented with acute-onset bloody stools and had similar episodes in the past. Technetium-99m pertechnetate scintigraphy demonstrates a focus of tracer uptake in the right mid abdomen on the 10-minute delayed image, with increasing conspicuity on the 20-minute delayed image (arrows). The findings were suggestive of Meckel’s diverticulum containing ectopic gastric mucosa and were confirmed at surgery.

**Figure S12. Bleeding jejunal lipoma.** **(a, b)** Axial noncontrast and arterial phase CT images demonstrate a fat-containing small-bowel lesion in the left upper quadrant with active contrast extravasation (arrows). A jejunal mass with active bleeding was seen on surgery confirmed on surgery and histopathology was consistent with a lipoma.

**Figure S13. Algorithmic approach to acute gastrointestinal bleeding.** UGIB: Upper gastrointestinal bleeding, EGD: Esophagogastroduodenoscopy, CTA: Multiphasic CT angiography, CA: Catheter angiography, LGIB: Lower gastrointestinal bleeding, SBB: Small bowel bleeding, Tc-99m–RBC Scan: Technetium-99m–labeled red blood cell scintigraphy.
